# Introducing Polyautoimmunity: Secondary Autoimmune Diseases No Longer Exist

**DOI:** 10.1155/2012/254319

**Published:** 2012-02-20

**Authors:** Adriana Rojas-Villarraga, Jenny Amaya-Amaya, Alberto Rodriguez-Rodriguez, Rubén D. Mantilla, Juan-Manuel Anaya

**Affiliations:** Center for Autoimmune Diseases Research (CREA), School of Medicine and Health Sciences, Universidad del Rosario, Kr 24 # 63 C 69 Third Floor, Bogotá, Colombia

## Abstract

Similar pathophysiological mechanisms within autoimmune diseases have stimulated searches for common genetic roots. Polyautoimmunity is defined as the presence of more than one autoimmune disease in a single patient. When three or more autoimmune diseases coexist, this condition is called multiple autoimmune syndrome (MAS). We analyzed the presence of polyautoimmunity in 1,083 patients belonging to four autoimmune disease cohorts. Polyautoimmunity was observed in 373 patients (34.4%). Autoimmune thyroid disease (AITD) and Sjögren's syndrome (SS) were the most frequent diseases encountered. Factors significantly associated with polyautoimmunity were female gender and familial autoimmunity. Through a systematic literature review, an updated search was done for all MAS cases (January 2006–September 2011). There were 142 articles retrieved corresponding to 226 cases. Next, we performed a clustering analysis in which AITD followed by systemic lupus erythematosus and SS were the most hierarchical diseases encountered. Our results indicate that coexistence of autoimmune diseases is not uncommon and follows a grouping pattern. Polyautoimmunity is the term proposed for this association of disorders, which encompasses the concept of a common origin for these diseases.

## 1. Introduction

Autoimmune diseases (ADs) have particular clinical characteristics and phenotypes depending on their nature (i.e., organ specific or systemic diseases). However, there is strong evidence that ADs share several clinical signs and symptoms, physiopathological mechanisms, and environmental and genetic factors, and this fact indicates that they have a common origin [1], which has been called the autoimmune tautology.

The clinical evidence of the autoimmune tautology highlights the cooccurrence of distinct ADs within an individual (i.e., polyautoimmunity) [1]. In an earlier paper, we described the foremost systematic literature review grouping all published cases of multiple autoimmune syndromes (MAS), defined by the presence of three or more well-defined ADs in a single patient, up until 2006. Initially, MAS was first mentioned by Pirofsky and Vaughn [2] and deeply described by Humbert and Dupond [3]. They provided a taxonomy for the cooccurrent phenotypes [4, 5]. MAS together with polyglandular autoimmune syndromes (PAS) II through IV, which are all MAS, represent the best example of polyautoimmunity [4]. Three basic, large clusters were found. Each of them had a predominant disease that was named the “chaperones” of autoimmunity, namely, autoimmune thyroid disease (AITD), Sjögren's syndrome (SS), and systemic lupus erythematosus (SLE). Study of the literature and clinical observation led to a similar clustering nomenclature which included the thyrogastric cluster and lupus-associated cluster [6, 7].

This coexistence of ADs in a single individual has lead researchers to consider different terms like autoimmune diathesis [8] or kaleidoscope of autoimmunity [9] both of which point to a common genetic background of ADs [10, 11]. The genetic basis of autoimmune clustering can depict part of the patterns of clustering across the spectrum of the implicated diseases [6].

Polyautoimmunity is also important for the current discussion because it may influence on the severity of ADs. In fact, some authors argue that there is a more severe presentation of a particular AD when polyautoimmunity is present [12–14], while others have found no influence or even a better prognosis in such cases [15–17].

In order to demonstrate one of the edges of autoimmune tautology, this study describes the presence of polyautoimmunity in four cohorts of ADs and analyses the main factors associated with its presence. In addition, an update of MAS cases is presented.

## 2. Material and Methods

### 2.1. Study Subjects

 Four previously published series of ADs patients were evaluated. All of them had cross-sectional designs analyzing the presence of polyautoimmunity in patients with SLE [18], rheumatoid arthritis (RA) [19], multiple sclerosis (MS) [20], and systemic sclerosis (SSc) [21]. All the patients were recruited from a multicenter cohort of ADs patients followed at the Center for Autoimmune Diseases Research (CREA) at the Universidad del Rosario in Bogota, Colombia. Patients fulfilled the American College of Rheumatology (ACR) criteria for the classification of SLE, SSc, and RA [22–24] and McDonald's criteria for MS [25]. The institutional review board approved the study design.

Each patient was evaluated by a rheumatologist or a neurologist depending on the case. The information on patient demographics and cumulative clinical and laboratory data was obtained by physical examination, interview, and chart review. All data were collected in an electronic and secure database.

There were 23 ADs investigated in the cohorts based on international validated criteria including autoimmune adrenal insufficiency (AAI: Addison's disease), alopecia areata (AA), autoimmune hepatitis (AIH), AITD, antiphospholipid syndrome (APS), biliary inflamatory disease including primary sclerosing cholangitis and primary biliary cirrhosis (BID), celiac disease (CD), demyelinating autoimmune diseases (DAD) including transverse myelitis (TM) and MS, dermatomyositis, polymyositis (DM/PM), inflamatory bowel disease including ulcerative colitis and Crohn disease (IBD), myastenia gravis (MG), pernicious anemia (PA), pemphigus (PF), psoriasis (Pso), RA, relapsing polychondritis (RePo), sarcoidosis (Sar), SS, SSc, SLE, type 1 diabetes mellitus (T1D), vasculitis (Vas), and vitiligo (VIT).

The presence of familial autoimmunity, including the presence of the same group of ADs evaluated in the search for polyautoimmunity, was estimated by interviewing the patients and, in most of the cases, by clinical evaluation of the affected family members as previously reported [26]. First-degree relatives (FDR) were defined as parents and siblings.

### 2.2. Systematic Literature Review

**Figure 1:**
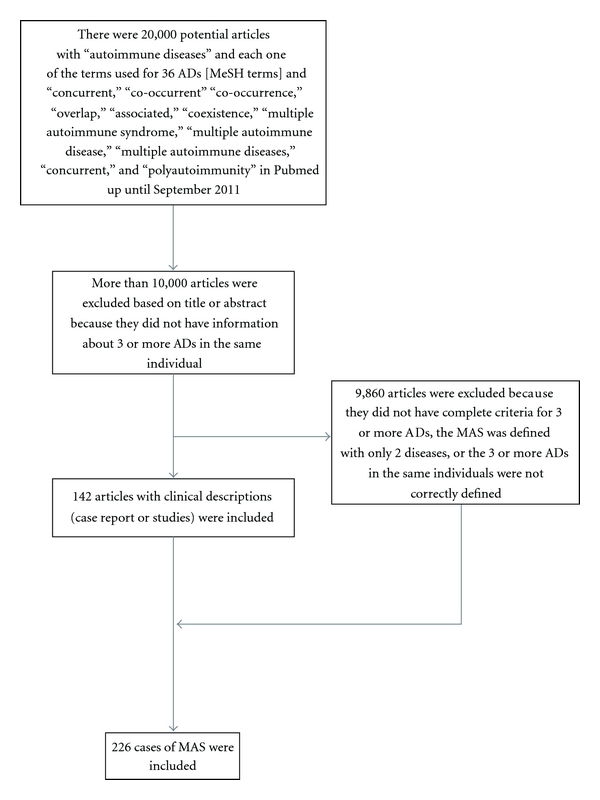
Flow chart of the systematic literature review. ADs: autoimmune diseases; MAS: multiple autoimmune syndrome.

An updated systematic literature review was done for all MAS cases reported from January 2006 to September 2011 (Figure 1). Publications were identified through a systematic search done by two independent experts in Pubmed. The only limits applied were Human. The [Majr] terms “multiple autoimmune diseases,” “multiple autoimmune syndrome,” “multiple autoimmune disease,” “polyautoimunity,” “co-occurrent,” “co-occurrence,” “coexistence,” “overlap,” “associated,” “concurrent”; [Mesh] terms: diabetes mellitus, type 1; antiphospholipid syndrome; lupus erythematosus, systemic; arthritis, rheumatoid; arthritis, juvenile rheumatoid; arthritis, psoriatic; spondylitis, ankylosing; spondylarthropathies; Sjögren's syndrome; Churg-Strauss syndrome; giant cell arteritis; microscopic polyangiitis; cryoglobulinemia; polyarteritis nodosa; Wegener granulomatosis; scleroderma, localized; scleroderma, systemic; scleroderma, diffuse; scleroderma, limited; dermatomyositis; colitis, ulcerative; Crohn disease; inflammatory bowel diseases; anemia, pernicious; thyroiditis, autoimmune; Hashimoto disease; Graves disease; celiac disease; hepatitis, autoimmune; liver cirrhosis, biliary; cholangitis, sclerosing; myasthenia gravis; multiple sclerosis; myelitis, transverse; polychondritis, relapsing; Addison's disease; purpura, thrombocytopenic, idiopathic; psoriasis; sarcoidosis; autoimmune gastritis; alopecia areata; autoimmune pancreatitis; pemphigus vulgaris; pemphigus bulloso; pemphigus foliaceous; vitiligo; autoimmune anemia.

Finally, the systematic literature review up to 2006 was checked and updated. The previous and new cases were compiled into a new table (see Supplementary Material available online at doi:10.1155/2012/254319).

### 2.3. Statistical Analysis

The prevalence of coexisting ADs was figured out separately by individual. The difference in the proportion of the associated ADs between two index conditions was calculated by chi-square, multinomial test corrected by Yate's continuity depending on the case. The degrees of freedom and Cramer's V were calculated.

A multivariate analysis was done for each of the series (SLE, MS, SSc, and RA) to identify factors associated with polyautoimmunity using logistic regressions models adjusted for age, gender, and duration of disease. Adjusted odds ratios (AORs) were calculated with 95% confidence intervals (CIs). A *P* value of less than 0.05 was considered significant. The Hosmer and Lemeshow Goodness-of-Fit Test was applied [27].

A hierarchical cluster procedure analysis was done to identify relatively homogeneous subgroups of variables based on selected cases with MAS reported in the systematic review of the literature. The reported cases from the previous systematic review [4] were computed with the results of the updated search. The objective of this analysis was to find out which ADs agglomerate more frequently. The cluster method implemented [28] was Single Linkage Sneath and the measure of similarity was Matching Coefficient. SPSS (V17 for Windows, Chicago, IL) software was used for all the analysis.

## 3. Results

### 3.1. Polyautoimmunity Patients

The 1,083 individuals studied included 335 SLE, 304 RA, 154 MS, and 290 SSc patients. There were 373 patients with polyautoimmunity (34.4%). The prevalence of polyautoimmunity was significantly different among the four ADs, being less frequent in MS (Table 1).

AITD was the most frequent coexisting AD and was associated with SSc in 23%  (*N* = 67) of the cases, RA in 21%  (*N* = 64), SLE in 17.9%  (*N* = 60), and MS in 9.1%  (*N* = 14). This was followed by SS which was associated with SSc in 14.8%  (*N* = 43) of the cases, SLE in 14%  (*N* = 47), RA in 11.8%  (*N* = 36), and MS in 2.6%  (*N* = 4). MAS was found in 11.6%  (*N* = 39), 9.7%  (*N* = 28), 5.3%  (*N* = 16), and 1.9%  (*N* = 3) of SLE, SSc, RA, and MS patients, respectively.

Factors significantly associated with polyautoimmunity are depicted in Table 2. Female gender was a shared factor that was significantly associated with polyautoimmunity in the four ADs. Familial autoimmunity was significantly associated with polyautoimmunity in SLE and SSc patients.

### 3.2. Systematic Literature Review

The flow chart for the systematic literature review and the articles included are shown in Figure 1. A total of 142 articles corresponding to 226 cases of MAS were included.

**Figure 2:**
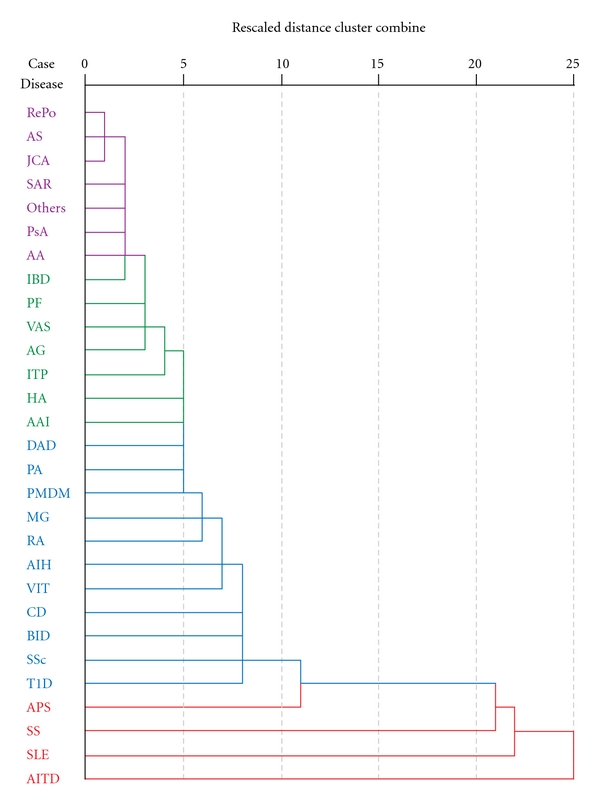
Cluster analysis dendogram. Each node represets a stage from the clustering process. There were four clusters. The most hierarchical was composed of four ADs. AITD: autoimmune thyroid disease (including thyroiditis, Hashimoto disease, Graves disease); SLE: systemic lupus erythematosus; SS: Sjögren's syndrome; APS: antiphospholipid syndrome; T1D: type 1 diabetes mellitus; SSc: scleroderma (including localized, systemic, diffuse, limited); BID: billiary inflammatory disease (including primary biliary cirrhosis, primary sclerosing cholangitis); CD: celiac disease; VIT: vitiligo; AIH: autoimmune hepatitis; RA: rheumatoid arthritis; MG: myasthenia gravis; PMDM: polymyositis/dermatomyositis; PA: pernicious anemia; DAD: demyelinating autoimmune diseases (including multiple sclerosis, transverse myelitis, optic neuromyelitis); AAI: autoimmune adrenal insufficiency (Addison disease); HA: autoimmune anemia; ITP: idiopathic thrombocytopenic purpura; AG: autoimmune gastritis; VAS: vasculitis (including Churg-Strauss syndrome, giant cell arteritis, microscopic polyangiitis, cryoglobulinemia, polyarteritis nodosa, Wegener granulomatosis); PF: pemphigus (including vulgaris, bulloso, foliaceous); IBD: inflammatory bowel disease (including ulcerative colitis, Crohn's disease); AA: alopecia areata; PsA: psoriasis (including psoriatic arthritis); SAR: sarcoidosis; JCA: juvenile chronic arthritis; AS: ankylosing spondylitis; RePo: relapsing polychondritis.

According to the dendogram (Figure 2), the most hierarchical AD in the MAS cases is represented by AITD followed by SLE and SS. Otherwise, the least representative diseases in the same context are juvenile chronic arthritis (JCA), ankylosing spondylitis (AS), and RePo. Although there were several articles about combined AIH and BID polyautoimmunity, the two were not close to each other on the dendogram nor did they show a suitable degree of agreement.

## 4. Discussion

Herein, we report one of the largest series of polyautoimmunity with an emphasis on its associated factors. Some authors had shown that so far the evidence suggesting that ADs tend to coexist within both individuals and families was anecdotal corresponding to the concept of autoimmune diathesis [29]. By grouping diverse ADs in the same patient (i.e., polyautoimmunity) including organ specific (i.e., MS) and systemic ADs, we have demonstrated that they are true associations as a part of the autoimmune tautology rather than the chance findings that were previous hypothesized [4].

Polyautoimmunity is a term that can group all the taxonomy terms referring to coexistence of well-defined ADs in a single individual because some of the terms previously used are confusing and exclude various associations. Polyautoimmunity was used by Sheenan and Stanton-King [30] for the first time while describing a patient with ITP, PA, AITD, SSc, pancreatic exocrine insufficiency, and CD before dying from vasculitic complications. The case they depicted corresponds to a typical MAS, which is already included in the term polyautoimmunity. Also, when patients fully develop two or more diseases simultaneously or sequentially, these diseases have frequently been classified as overlap syndromes; some of these were frequent enough to have been given names like rhupus and sclerodermatomyositis [31]. In another case, some authors have historically postulated that mixed connective tissue disease (MCTD) is a very homogeneous entity with shared clinical manifestations rather than shared diseases or autoantibodies [32, 33], while others have not. The existence of MCTD as a distinct disease entity has been a matter of controversy among researchers since it was first described [34, 35]. In fact, the coexistence of several sets of classification criteria for MCTD indicates how difficult it is to give a precise definition of the disease [33]. In addition, some patients will develop SLE, SSc, or RA, during the course of MCTD, and some will present with a longstanding MCTD [36]. In real-life conditions, searching for the specific phenotypes (antibodies and clinical) over the course of disease and constantly looking for associated ADs, including organ specific and systemic, are more useful for developing an exact description of polyautoimmunity than taxonomic discussions.

The fact that many ADs share a similar underlying pathology and have a tendency to cluster supports the involvement of shared susceptibility genes and similar molecular mechanisms. In fact, recent studies have identified several common genes associated with multiple ADs supporting the presence of autoimmunity genes as part of the autoimmune tautology [10, 37, 38].

Familial autoimmunity and female gender were confirmed as risk factors for polyautoimmunity. Female gender was a shared factor associated with polyautoimmunity in the four index conditions here studied. This fact gives us a glimpse of one facet of the shared commonalities between ADs. The majority of ADs predominate in females [39] and constitute a leading cause of death among young and middle-aged women [40]. In searching for a reason behind female predominance, most attention has focused on hormonal changes while other factors have included genetic differences, both direct (i.e., influence of genes on sex chromosomes) and indirect such as microchimerism, as well as gender differences in lifestyle [39, 41]. Our results support previous studies including a meta-analytic approach demonstrating that most of the patients in 54 studies quantifying the coexistence of ADs among 4 selected ADs were female [29]. In addition, some authors have shown in specific ADs that women are distinguished from men by higher frequencies of concurrent immune diseases [42, 43].

AITD was the most frequent polyautoimmunity found in our series of 1,083 patients. This finding was supported by the analysis of the systemic literature review and depicted in the dendogram (Figure 2) where AITD was the main “chaperon” of autoimmunity. AITD has been described as the most prevalent AD as well as being associated with other organ-specific and non-organ-specific ADs [44].

Possible explanations for the relationship of these ADs include (a) immunomodulatory effects of antithyroid antibodies, (b) molecular mimicry between thyroid and disease-specific epitopes, and (c) a genetic link between antithyroid autoimmunity and the susceptibility to AD [45]. In population-based database studies, other authors have demonstrated that AITD is frequently associated with other ADs [46]. All of this information indicates that AITD is clinically important in the context of autoimmunity and it is mandatory for screening patients with hypothyroidism or hyperthyroidism symptoms for the autoimmune etiology when there is suspicion of the coexistence of AITD with another AD [44].

The prevalence of SS was demonstrated to be high and in fact the second most frequently associated AD in our series as well as in the MAS cases through the literature review. Many authors have recognized that it is quite difficult to categorize concomitant SS as primary or secondary, and there is disagreement about this issue in the literature [47]. Other authors believe that salivary changes in patients with an AD (i.e., SLE) might reflect a multisystem presentation of the disease [48]. Regarding the association of SS with other ADs, some authors have argued that the etiopathogenic mechanism for the simultaneous or sequential development of multiple ADs in one individual is not well understood [49]. The concept is more developed nowadays, and the idea that common genetic backgrounds and additional immunogenetic, environmental, or hormonal factors are responsible for the formation of subsets of AD clusters is becoming more established.

We previously evaluated the prevalence of SS in a large series of patients with SLE (*n* = 969) and the potential risk factors for this association [50]. SS patients fulfilled the American-European classification criteria (the presence of anti-Ro antibodies or a positive minor salivary gland biopsy was mandatory). There were 9.3% patients with SS, 42% had familial autoimmunity, of which 7% had familial SS as compared to 2% in the group of SLE without SS. The factors significantly associated with SS in SLE were familial SS, anti-La and anti-Ro antibodies, as well as pulmonary involvement. Anti-Sm antibodies and Colombian origin (i.e., ethnicity) were protective factors. Our results together with other series [51, 52] using similar strict classification criteria indicate that the prevalence of SS in SLE is close to 10%. SLE-SS appears to constitute a subgroup of patients with distinct clinical, serologic, pathologic, and immunogenetic features, in whom SS is expressed as an associated entity and is largely similar to what has been called primary SS [52]. Clinical and immunological factors observed in our study might serve as predictors for this association. Because variations in both additive and nonadditive genetic factors and the environmental variance are specific to the investigated population, family history of autoimmunity and patient origin are important characteristics to be considered.

While the currently most accepted classification criteria [53] designate these cases as “secondary” SS, the terms “overlapping” or “associated” SS are frequently used in the literature to describe them [49]. We consider these terms to be confusing and propose that SS always be taken into account and properly investigated in patients diagnosed with any AD because of the high possibility of the presence of the concomitant, well-defined phenotypes as demonstrated here in the context of polyautoimmunity or as demonstrated previously with a prevalence near to 10% in SLE patients when strict classification criteria are used.

It is of interest that the primary/secondary designation for classification of APS was introduced by rheumatologists who already used the primary/secondary terms to differentiate subgroups of patients with SS. In introducing the primary APS (PAPS) subgroup, it was unclear whether one would expect that the clinical features, disease course, or management of patients would be different based on their subclassification [54]. In fact, the international (Sydney) consensus statement on an update of the classification criteria for definite APS [55] advises against using the term “secondary” APS. They did not find differences in the clinical consequences of antiphospholipid antibodies among patients in these two categories (Evidence Level I). They state most patients with the so-called secondary APS have SLE. However, they discuss that it is unknown if APS and SLE are two diseases coinciding in an individual, if underlying SLE offers a setting for the development of APS, or if APS and SLE represent two elements of the same process [55]. Some cases with “secondary” APS are classified as lupus like disease (LLD). The Sydney consensus raised up that the interface between SLE, LLD, and APS merits further consideration. Finally, the consensus states that rather than distinguishing between patients with PAPS and secondary APS, documenting the coexistence of SLE (or other disease) is more advantageous for classification and that the disorder associated with APS, such as SLE, be listed; hence, one would report “APS associated with SLE” or “APS associated with rheumatoid arthritis” rather than “secondary APS” [54]. Studies showing no significant differences between PAPS and SAPS were cited. Patients with APS plus SLE and PAPS have similar clinical profiles, although heart valve disease, hemolytic anemia, low C4 levels, and neutropenia seem to be more common in patients with APS plus SLE [56].

Indeed evidence that there are any differences in presentation or course of PAPS versus SAPS is not persuasive [55, 56], and this has led to the suggestion that PAPS/SAPS designations be replaced by APS and “APS associated with the name of the autoimmune disease.” It might suggest that such a distinction exists if there are differences in clinical complications, the timing of these complications, prognosis, or frequency of positive anticardiolipin, lupus anticoagulant, or other autoantibody tests. Studies that have addressed this question have found no difference in any of these parameters [57, 58]. As an instance, some authors have compared intima-media thickness (IMT), arterial stiffness, and presence of plaques in APS patients and controls. A significant difference was found between IMT, arterial stiffness, and the presence of plaques in patients and controls, but no differences in these parameters were found between patients with primary APS and those with secondary APS [59]. Additional arguments have been raised for some authors [60, 61] who have explored the concept of an intermediate APS (having at least one but less than four of the 11 criteria for SLE) and did not found differences between PAPS, intermediate, and the so-called secondary APS when comparing the prevalence of the thrombotic or pregnancy manifestations.

The true prevalence of the development of PAPS in SLE will require decades of followup for this determination. The distinction between PAPS and SAPS can be difficult and at times seems a rather artificial convention [60]. It may be underestimated by some studies that have a follow-up period that is shorter than the interval between PAPS and SLE diagnoses noted in most case reports [60]. We agree on the proposition of the Sydney committee [55] against using the term “secondary” APS and encourage clinicians to follow adequately the patients and searching for specific phenotypic characteristic to classify patients as having polyautoimmunity.

Results with respect to the severity of the disease in patients with polyautoimmunity are not unanimous. In the case of associated SS, some authors have demonstrated as we did previously [17] that there is no influence on the course of the disease. Some have found that the appearance of SS in RA patients has no relationship with RA duration or activity [62]. Others demonstrated that the subset of patients with SLE and SS has a distinct clinical and laboratory phenotype with a lower frequency of renal disease and anti-dsDNA antibodies [63]. This has not been true for other examples of polyautoimmunity when there is a severe presentation of the diseases as is the case of associated ADs in MG patients with a severe presentation [12], a severe clinical onset of T1D and increased prevalence of other ADs in children with CD diagnosed before T1D [13], and a severe SLE compromise when associated with vasculitis [14].

In conclusion, we suggest searching for well-defined phenotypes by looking for clusters of ADs in the same individual. It is our contention that the term “secondary diseases” should not longer be used because it detracts from the reality that these patients have two or more well-established ADs sharing the same etiopathogenesis [64]. Our results indicate that coexistence of ADs is not uncommon and follows a grouping pattern. Polyautoimmunity is the term proposed for this association of disorders, which encompasses the concept of a common origin for these diseases.

## Supplementary Material

This table contains the detailed results about systematic literature review up to 2011: 142 articles with 226 cases of MAS were reported. In the first page (second column) there is the corresponding author's name and following there are the columns with the abbreviation of the specific AD. In each row the symbol characterizes each one of the disease found per case. In the second page, there is the complete article's name and the link to Pubmed for each reference.

## Figures and Tables

**Table 1:** Polyautoimmunity in 1,083 patients with four index autoimmune diseases.

	SLE		RA		MS		SSc		chi	df	*P*	Cramer's V
	*N*	%	*N*	%	*N*	%	*N*	%				
N	335	30.9	304	28.1	154	14.2	290	26.8				
Polyautoimmunity	136	40.6	98	32.2	21	13.6	118	40.7	40.81	3	0.001	0.19
AITD	60	17.9	64	21.1	14	9.1	67	23.1	14.63	3	0.0022	0.11
SS	47	14.0	36	11.8	4	2.6	43	14.8	16.4	3	0.0009	0.12
VIT					2	1.3						
APS	48	14.3	8	2.6					25.83	1	0.001*	0.2
Primary biliary cirrosis							15	5.2	—	—	—	—
MAS	39	11.6	16	5.3	3	1.9	28	9.7	17.99	3	0.0004	0.12

SLE: systemic lupus erythematosus; RA: rheumatoid arthritis; MS: multiple sclerosis; SSc: systemic sclerosis; AITD: autoimmune thyroid disease; SS: Sjögren's syndrome; VIT: vitiligo; APS: antiphospholipid syndrome; MAS: multiple autoimmune syndrome; chi: chi-square test; df: degree freedom; *P*: *P* value; *Yates chi-square.

**Table 2:** Significant factors associated with polyautoimmunity.

	SLE		RA		MS		SSc	
	AOR; CI95%	*P*	AOR; CI95%	*P*	AOR; CI95%	*P*	AOR; CI95%	*P*
Female gender	2.3; 1.03–5.15	0.043	1.8; 1.22–6.31	0.015	8.5; 1.02–70.8	0.048	9.08; 2.09–39.3	0.003
Familial autoimmunity	1.61; 1.14–2.28	0.007	NS		NS		2.62; 1.24–5.54	0.01
Articular Involvement	2.02; 1.26–3.23	0.003	NS		NE		NS	
Anti-Ro positivity	1.54; 1.10–2.16	0.013	NS		NE		NS	
Cardiovascular disease	NS		2.2; 1.17–3.94	0.014	NE		NE	
ANAs	NS		2.0; 1.08–3.84	0.027	NS			
SSEP	NE		NE		10.86; 1.31–89.6	0.027	NE	

SLE: systemic lupus erythematosus; RA: rheumatoid arthritis; MS: multiple sclerosis; SSc: systemic sclerosis; AOR: adjusted odd ratio; CI95%: confidence interval; ANAs: antinuclear antibodies; SSEP: somatosensory evoked potentials; NS: nonsignificant; NE: not evaluated.
